# Brachiaria Grasses (*Brachiaria spp*.) harbor a diverse bacterial community with multiple attributes beneficial to plant growth and development

**DOI:** 10.1002/mbo3.497

**Published:** 2017-06-21

**Authors:** Collins Mutai, Joyce Njuguna, Sita Ghimire

**Affiliations:** ^1^ Biosciences eastern and central Africa‐International Livestock Research Institute (BecA‐ILRI) Hub Nairobi Kenya

**Keywords:** ACC deaminase, antifungal activity, hydrogen cyanide, indole‐3‐acetic acid, phosphate solubilization, plant beneficial properties, siderophore production

## Abstract

Endophytic and plant‐associated bacteria were isolated from plants and rhizoplane soil of naturally grown Brachiaria grasses at International Livestock Research Institute in Nairobi, Kenya. Eighty‐four bacterial strains were isolated from leaf tissues, root tissues, and rhizoplane soil on nutrient agar and 869 media. All bacterial strains were identified to the lowest possible taxonomic unit using 16S rDNA primers and were characterized for the production of Indole‐3‐acetic acid, hydrogen cyanide, and ACC deaminase; phosphate solubilization; siderophore production; antifungal properties; and plant biomass production. The 16S rDNA‐based identification grouped these 84 bacterial strains into 3 phyla, 5 classes, 8 orders, 12 families, 16 genera, and 50 unique taxa. The four most frequently isolated genera were *Pseudomonas* (23), *Pantoea* (17), *Acinetobacter* (9), and *Enterobacter* (8). The functional characterization of these strains revealed that 41 of 84 strains had a minimum of three plant beneficial properties. Inoculation of maize seedlings with *Acinetobacter* spp., *Microbacterium* spp., *Pectobacterium* spp., *Pseudomonas* spp., and *Enterobacter* spp. showed positive effects on seedling biomass production. The ability of Brachiaria grasses to host genetically diverse bacteria, many of them with multiple plant growth‐promoting attributes, might have contributed to high biomass production and adaptation of Brachiaria grasses to drought and low fertility soils.

## 1 INTRODUCTION

Brachiaria is an important constituent of Savannah grassland ecosystem that has been supporting millions of African herbivores for thousands of years (Kelemu et al., 2011), and they consist of about one hundred documented species; several of which are used as cultivated pastures across the tropics. Brachiaria is the most extensively grown tropical forage in Latin America, Asia, South Pacific, and Australia with an estimated acreage of 99 million hectares in Brazil alone (Jank, Barrios, do Valle, Simeão, & Alves, 2014). Recently, there has been considerable interest in Brachiaria grasses in Africa, and several initiatives are ongoing to promote Brachiaria to support the emerging livestock industry in the region, especially in the dry season (Maass et al., 2015). Brachiaria grasses have several desirable traits that include: adaptation to marginal soils, water stresses and shade tolerance, high biomass production potential, ability to sequester carbon, increased nitrogen use efficiency through biological nitrification inhibition (BNI), and subsequently the ability to reduce greenhouse gas emissions and ground water pollutions (Fisher & Kerridge, 1996; Fisher et al., 1994; Rao, Kerridge, & Macedo, 1996; Subbarao et al., 2009). Brachiaria, being a highly palatable and nutritious forage, increases livestock productivity. Moreover, Brachiaria is an important ecological agent with significant roles in soil improvement and erosion control. Despite the plethora of desirable attributes and high biomass production potential of 30 t/ha, the on‐farm productivity of Brachiaria in Africa is quite low.

The global demand for livestock product is projected to increase by 70% in 2050 due to growing population, rising affluence, and urbanization (FAO, 2016). Forages are the main component of livestock feeds accounting for 60–80% of livestock production costs (Ademosun, 1976). The economic production of forages can be achieved by minimizing the production costs and/or by closing the yield gaps. The cultivation of high yielding forage species that require expensive inputs such as irrigation, fertilizers, and other agrochemicals is not a feasible option for the smallholder farmers in Africa. Therefore, the development of low‐input forage production technologies that maximize the use of local resources with concomitant decrease in expensive external inputs is necessary to meet the growing demand for quality forages.

Endophytic and plant growth‐promoting rhizobacteria (PGPR) are known to provide several fitness benefits to plant hosts. These benefits include nitrogen fixation (Bahulikar, Torres‐Jerez, Worley, Craven, & Udvardi, 2014; James, 2000), the production of auxins, cytokinins, and gibberellins (García de Salamone, Hynes, & Nelson, 2001; Gutierrez‐Manero et al., 2001; Taghavi et al., 2009), suppression of the ethylene production by 1‐aminocyclopropane‐1‐carboxylate (ACC) deaminase activity (Taghavi et al., 2009; Zhang et al., 2011), alteration of sugar‐sensing mechanisms in plants (Goddijn & Smeekens, 1998; Taghavi et al., 2009), solubilization of mineral phosphorous to a form that is readily available to plants (Otieno et al., 2015; Turan et al., 2012), and synthesis of siderophores (Kloepper, Leong, Teintze, & Schroth, 1980; Rungin et al., 2012) and other low molecular mass compounds or enzymes that can modulate plant growth and development (Davison, 1988; Lambert & Joos, 1989). Endophytic and PGPR also provide benefit to host plants by preventing or suppressing plant pathogens through competition for niche and nutrients, by antibiosis, through the production of hydrolytic enzymes and through induced systemic resistance (Compant, Duffy, Nowak, Clément, & Barka, 2005; Mendes et al., 2011; van der Lelie et al., 2009). A model system involving plant bacterial association (poplar host and *Enterobacter* spp.) is well recognized for a variety of fitness enhancement on poplar and other plant species (Taghavi et al., 2010). Endophytic and PGPR seem to exist in most, if not all, higher plant species (Mastretta et al., 2006; Wu, Wan, Shengchun Wu, & Wong, 2012). The utilization of endophytes and PGPR bacteria is, therefore, a feasible strategy for enhancing the productivity of a wide range of plant species, but this is severely constrained by a limited understanding of these microbes in different hosts. The objectives of this study were as follows: (1) to catalog cultivable bacterial endophytes and PGPR of the important tropical forage *Brachiaria* spp., (2) to characterize the roles of these endophytes and PGPR on plant growth and development, and (3) identify candidate microbes for potential use in the commercial cultivation of Brachiaria grasses in Sub‐Saharan Africa.

## 2 EXPERIMENTAL PROCEDURES

### 2.1 Sample collection

Leaves, roots, and rhizoplane soil were collected from 30 different and apparently healthy looking, Brachiaria plants growing in wild at the farm of the International Livestock Research Institute (ILRI) in Nairobi, Kenya, for bacterial isolation. After collecting samples for bacterial isolation, plants were maintained in field at ILRI Campus.

### 2.2 Surface sterilization

The Brachiaria leaves and roots samples were processed and sterilized as described by Taghavi et al. (2009) with slight modification. Briefly, samples were thoroughly washed in running tap water then cut into small (3–4 cm) pieces, surface sterilized in 70% ethanol for 1 min, and subsequently in 1.2% sodium hypochlorite (NaOCl), amended with two drops of Tween‐20 per 100 ml solution, for 10 min for leaf and 20 min for root samples. The samples were rinsed three times in sterile distilled water and blot dried in between sterile paper towels.

### 2.3 Bacterial isolation

One gram of surface‐sterilized plant samples, cut into 2–3 mm pieces, was macerated in 9 ml of 10 mmol/L magnesium sulfate (MgSO_4_) solution using a sterile mortar and pestle and the suspension diluted serially. Rhizoplane soil was collected from the freshly collected roots, 1 g of soil was added to 9 ml of 10 mmol/L MgSO_4_, mixed vigorously for 5 min, allowed to settle for 5 min, and the supernatant was collected for serial dilution. A total volume of 100 μl samples from serial dilutions was plated on nutrient agar and 869 media, incubated at 28^°^C for up to 3 days and the emergent colonies picked and purified through three subsequent single‐colony subcultures.

### 2.4 DNA extraction, 16S rRNA gene amplification and sequencing

Bacterial genomic DNA was extracted using PrepMan^®^ reagent (Applied Biosystems) according to the manufacturer's instructions. The 16S rRNA gene was amplified using primer pairs 27F (5′‐AGAGTTTGATCCTGGCTCAG‐3′) and 1492R (5′‐GGTTACCTTGTTACGACTT‐3′) (Frank et al., 2008). The PCR was performed using AccuPower^®^ PCR PreMix (Bioneer) in 25 μl reactions under cycling conditions consisting of an initial denaturation at 95^°^C for 2 min followed by 30 cycles of denaturation at 94^°^C for 45 s, annealing at 57^°^C for 45 s and extension at 72^°^C for 45 s; and a final extension at 72^°^C for 10 min. Amplification products were run on 1% agarose‐0.5XTris‐Borate‐EDTA gels and PCR products were purified using a QIAquick PCR Purification Kit (Qiagen). Purified PCR products were sequenced using the ABI 48‐capillary 3730 DNA Analyzer (Applied Biosystems).

### 2.5 Bioinformatics analysis

The raw sequences were processed and analyzed using the CLC Genomics Workbench 7.0.3 (http://www.clcbio.com), and molecular identities of the bacterial strains obtained using the SeqMatch tool on the Ribosomal Database (Cole et al., 2014). In addition, BLAST analysis was performed on the NCBI database and results from the two databases compared. The nucleotide sequences from test strains were aligned, after eliminating gaps and missing data, and unique taxa were identified by sequence homology search. For the phylogenetic analysis, the unique sequences, eight reference sequences, and one out‐group sequence were included. The tree was generated using the tree builder tool on the RDP database (Cole et al., 2014) and the trees were compared to those generated using MEGA version 6 (Tamura, Stecher, Peterson, Filipski, & Kumar, 2013).

### 2.6 Biochemical characterization of bacterial strains

Bacterial strains isolated from Brachiaria leaves, roots, and rhizoplane soil were tested for several biochemical properties beneficial to plant growth and development adopting the procedures described below.

### 2.7 Indole‐3‐acetic acid (IAA) production

Bacterial strains were grown in 1/10th strength 869 broth supplemented with 0.5 g per liter of L‐tryptophan at 28^°^C for 5 days at 150 rpm in dark. After growth the number of bacterial cells was estimated using a spectrophotometer at 600 nm with one OD value considered to be equivalent to 1.0 × 10^8^ bacterial cells per milliliter of culture media. The cultures were subsequently centrifuged at 2147 × g for 10 min and one volume of the clarified supernatant added with two volumes of Salkowski reagent (Mayer, 1958) and incubated for 35 min at room temperature. Development of a pink color, an indication of IAA production, was quantified by measuring absorbance at 535 nm.

### 2.8 Hydrogen cyanide (HCN) production

Qualitative HCN detection was performed using Lorck's alkaline picrate assay (Alstrom & Burns, 1989; Lorck, 1948). Bacterial strains were cultured in nutrient agar supplemented with 4.4 g of glycine per liter and poured onto 24‐well culture plates. HCN production was detected by placing Whatman No.1 paper discs soaked in a solution of 0.5% picric acid in 2% sodium carbonate a few millimeters above the surface of inoculated media in each well. The plates were incubated at 28^°^C for 4 days. The change in filter paper color from yellow to light brown, brown, and reddish‐brown was indicative of weak, moderate, and high levels of HCN production, respectively.

### 2.9 1‐aminocyclopropane‐1‐carboxylic acid (ACC) deaminase production

Bacterial strains were tested for ACC deaminase production as described previously (Ali, Sandhya, & Rao, 2014). Briefly, bacterial strains were grown in Dworkin and Fosters ACC minimal salts supplemented with ACC as a sole nitrogen source. Media without ACC and with nitrogen source (2 g/L of (NH4)_2_SO4) were used as controls. The ability of a strain to grow in media with ACC as sole source of nitrogen, and no growth of the same strains in nitrogen‐free media, was indicative of ACC deaminase production.

### 2.10 Phosphate solubilization

The ability of bacterial strains to solubilize phosphate was determined by spotting 10 μl of freshly prepared bacterial cells onto NBRIP media (Mehta & Nautiyal, 2001). Inoculated plates were incubated at 28^°^C for 2–3 days. Formation of clear halos around the colony is indicative of phosphate solubilization and the test strains were scored as either positive or negative for the ability to solubilize mineral phosphorus.

### 2.11 Siderophore production

The ability of bacterial strains to produce siderophores was determined using Chrome‐Azurol (CAS) media as described previously (Vellore, 2001). Briefly, bacterial strains were grown overnight at 28^°^C on a shaker in two variants of modified Fiss minimal medium (5.03 g/L KH_2_PO_4_, 5.03 g/L L‐asparagine, 5.0 g/L glucose, 40 mg/L MgSO_4_, 100 μg/L MnSO_4_, and 500 μg/L ZnCl_2_). Iron‐restricted modified Fiss minimal medium and high iron modified Fiss minimal medium were prepared by adding 139 μg/L FeSO_4_ (5 μmol/L) and 5.56 mg/L FeSO_4_ (20 μmol/L) to the final media, respectively. Siderophore production was examined by loading 60 μl of culture supernatant, obtained after centrifugation at 15339 × g for 2 min, and filtration through 0.2 μm Millipore filters, into wells in the CAS media. Plates were incubated at 28^°^C for 3–5 days. The development of yellow or orange halos around the inoculated wells is indicative of siderophore production and the strains were scored as positive or negative for siderophoregenesis.

### 2.12 Antifungal activities

Bacterial strains were tested for the ability to inhibit the growth of seven isolates of plant pathogenic fungi: *Aspergillus flavus* (F006)**,** *Aspergillus flavus* (F023), *Nigrospora oryzae* (F025), *Fusarium equiseti* (F005), *Nigrospora sphaerica* (F010), *Phoma herbarum* (F020), and *Magnaporthe grisea* (MG001). One 5 mm × 5 mm agar block from the edge of each actively growing fungal colony (7–10 days old) was cut and transferred onto a sterile 1.5 ml Eppendorf tube containing 300 μl of sterile water and ground into a fine suspension. A total volume of 100 μl of the mycelial suspension was added to 100 ml Potato Dextrose Agar (PDA) at 55^°^C, thoroughly mixed, poured onto 90 mm Petri dish, and allowed to set at room temperature. Thereafter, 10 μl of fresh bacterial cells of each test bacterial strain was spotted onto the plates and incubated at 28^°^C for 7–10 days to allow the fungi and bacteria to grow together. Inhibition of fungal growth around the bacterial colonies was used as an indication of antifungal activity of test strain. The size of the zone of inhibition was used as a measure of the degree of antifungal activity.

### 2.13 Screening for plant growth promotion

Twenty‐eight of 84 bacterial strains with varied functional properties and water inoculated control were tested in a greenhouse experiment for biomass production using a maize seedling system. One additional bacterial strain (CSB_B007) that showed positive response to plant growth in an earlier study was included as positive control. Five maize seeds were planted in gallon pots filled with heat‐sterilized mixture of loam virgin forest soil and animal manure (ratio of 3:1 v/v), thinned to three seedlings per pot after a week of emergence, and each seedling was inoculated with 2 ml of bacterial suspension containing 1.0 × 10^8^ bacterial cells per ml, while control plants were inoculated with 2 ml of sterile water. Each treatment had a total of six seedlings and the experiment was repeated twice. Inoculated plants were maintained in a greenhouse (daily mean temperature of between 20 and 23^°^C and mean relative humidity of 51–67%, and 12 hrs day length) for 3 weeks. Plants were harvested, separated into shoot and root, dried at 60^°^C for a minimum of 72 hrs, and weighed to determine biomass. The biomass data were analyzed using Genstat (VSN International, 2013). Dry biomass of shoots, roots, and the sum of the two was used as response variables, with the experiment and bacterial strain treatment as predictors. The treatment‐wise means for root, shoot, and total biomass production were computed and compared using standard error of means. Because the experiments differed significantly (*p* > .05) for biomass productions, data for the two experiments were analyzed separately.

## 3 RESULTS

### 3.1 Bacterial isolation and molecular identifications

**Figure 1:**
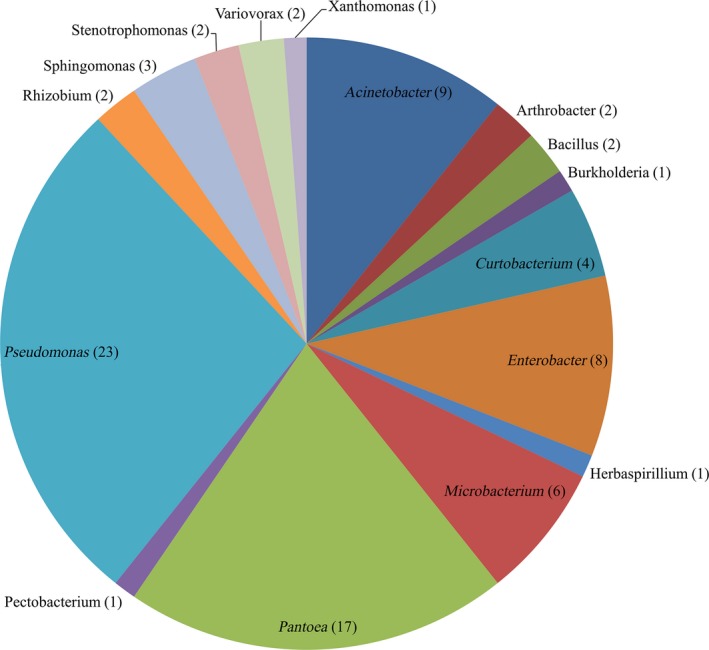
The frequency of bacterial genera isolated from the roots, leaves, and rhizoplane soil of Brachiaria grasses grown in a natural habitat. The number in parenthesis indicates the number of isolated bacterial strains belonging to each corresponding genus

A total of 84 bacterial strains were isolated from Brachiaria leaves, roots, and rhizoplane soil. The laboratory IDs of these strains are presented in supplementary table S1. The number of bacterial strains isolated from leaves, roots, and rhizoplane soil was 27, 26, and 31, respectively. The 16S rDNA sequences were generated for all the tested strains, and homology search in RDP and NCBI databases revealed them into three phyla, five classes, eight orders, twelve families, and sixteen genera (Table 1; Figure 1). Based on 16S rDNA sequences, these 84 bacterial strains belonged to 50 unique taxa. The sequences of these 50 taxa were submitted to NCBI and are published in the Genbank ^®^ Database under accession numbers from KU725918 to KU725967.

**Table 1:** Grouping of 84 bacterial strains isolated from Brachiaria tissues and rhizoplane soil at different taxonomic levels

Phylum	Class	Order	Family
Proteobacteria (70)	Alphaproteobacteria (5) Betaproteobacteria (4) Gammaproteobacteria (61)	Burkholderiales (4) Enterobacteriales (26) Pseudomonadales (32) Rhizobiales (2) Sphingomonadales (3) Xanthomonadales (3)	Burkholderiaceae (1) Comamonadaceae (2) Enterobacteriaceae (26) Moraxellaceae (9) Oxalobacteraceae (1) Pseudomonadaceae (23) Rhizobiaceae (2) Sphingomonadaceae (3) Xanthomonadaceae (3)
Actinobacteria (12)	Actinobacteria (12)	Actinomycetales (12)	Micrococcaceae (2) Microbacteriaceae (10)
Firmicutes (2)	Bacilli (2)	Bacillales (2)	Bacillaceae (2)

**Figure 2:**
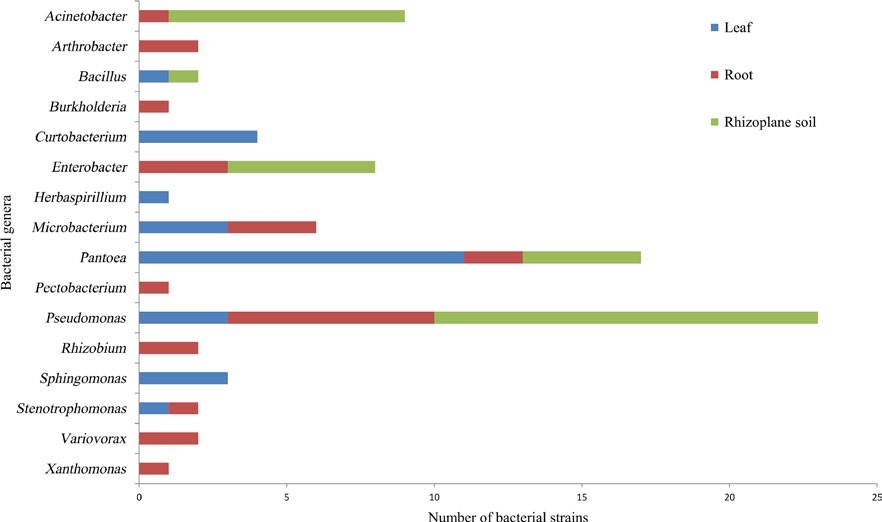
Distribution of bacterial genera in leaves, roots, and rhizoplane soil of naturally growing and apparently healthy looking *Brachiaria* grasses

The three most frequently isolated genera were *Pseudomonas*,* Pantoea*, and *Acinetobacter* with the frequency of 23, 17, and 9 of the total isolates, respectively (Figure 1). *Pseudomonas* and *Pantoea* were isolated from all the three sources while *Acinetobacter* was isolated from root and rhizoplane soil. Similarly, *Bacillus*,* Microbacterium*,* Stenotrophomonas*, and *Enterobacter* had two different origins, while *Arthrobacter*,* Burkholderia*,* Pectobacterium*,* Rhizobium*,* Variovorax*, and *Xanthomonas* were isolated exclusively from root samples. *Curtobacterium*,* Herbaspirillium*, and *Sphingomonas* were isolated exclusively from leaf samples (Figure 2).

### 3.2 Phylogenetic relationships among bacterial isolates

**Figure 3:**
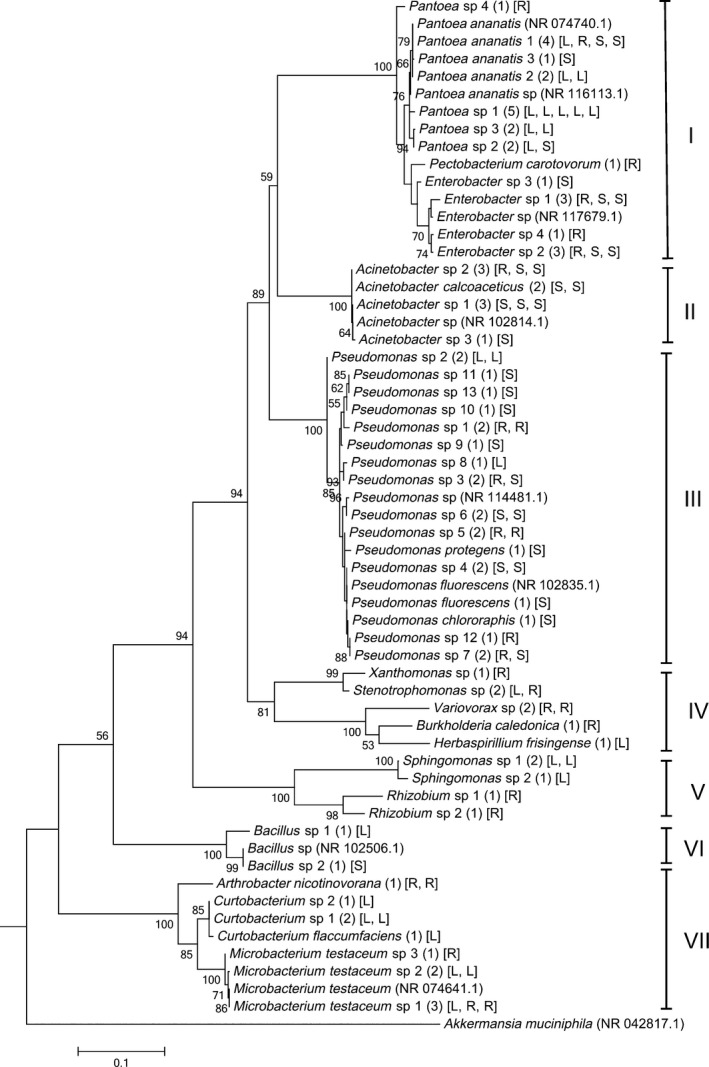
Phylogenetic analysis of 50 representative bacterial taxa isolated from Brachiaria grasses inferred using the Maximum Likelihood method based on the Tamura three‐parameter model. Eight reference strains and one out‐group were included in the analysis (indicated with the accession number in parenthesis). Each taxon represents 1–5 strains as shown in the parentheses with their sources (L= leaves, R= roots, and S= rhizoplane soil). The tree is drawn to scale, with branch lengths measured in the number of substitutions per site. All positions containing gaps and missing data were eliminated; with a total of 1,051 positions in the final dataset

The 16S rRNA gene sequence‐based neighbor‐joining cladogram grouped the 50 representative bacterial taxa into seven major clades with a strong bootstrap support (Figure 3). Majority of strains (72.6%) belonged to three major clades, that is, clade I, III, and VII. Clades I and III consisted of strains from all three sources; clades IV, V, and VII had strains exclusively from roots and leaves; clade II had strain from roots and soils only; and clade VI had strains from leaves and soils only. Clade IV had the highest diversity at the genus level.

### 3.3 Biochemical characterization of bacteria

All 84 bacterial strains from leaves, roots, and rhizoplane soil were tested for six biochemical properties: production of Indole‐3‐acetic acid, hydrogen cyanide, and ACC deaminase; phosphate solubilization; siderophore production; and antifungal activities (Figure S1 A‐F). The biochemical profiles of individual tested strains are presented in supplementary Table S1.

**Figure 4:**
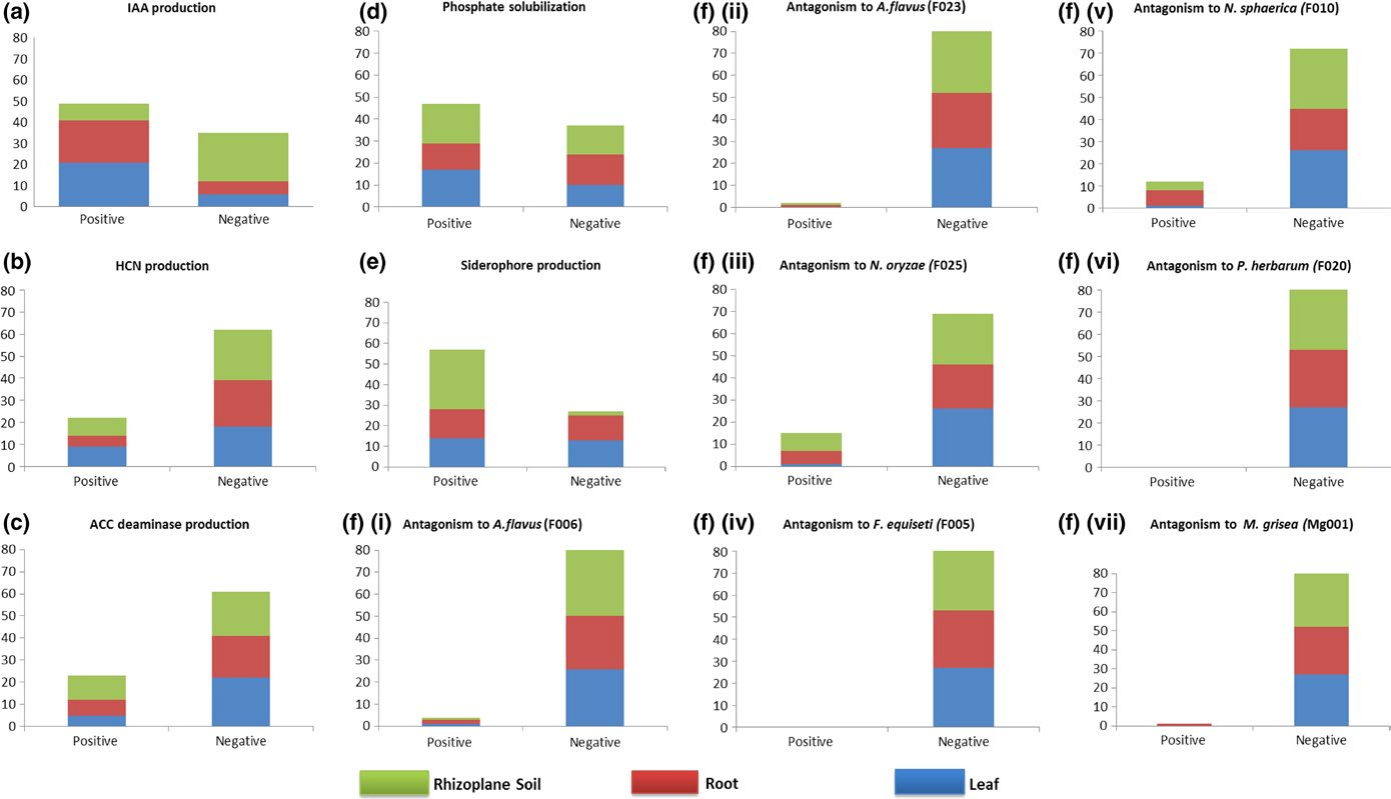
Biochemical characterization of endophytic and plant‐associated bacteria of Brachiaria grasses. Isolated strains were tested for various plant growth‐promoting attributes including auxin (IAA) production (a), cyanogenesis (b), ACC‐deaminase production (c), phosphate solubilization (d), siderophoregenesis (e), and antifungal activity (Fi‐vii). The y‐axes represent the number of bacterial strains characterized for each plant beneficial property

Forty‐nine of the 84 bacterial strains were positive for IAA production (Figure 4a). Majority of these positive strains were isolated from leaf (*n* = 29) and roots (*n* = 20). On the other hand the majority of the strains (23 of 35) that tested negative for IAA production were from the rhizoplane soil. Twenty‐two of the tested strains were positive for cyanogenesis. Both the HCN‐negative and HCN‐positive strains were isolated from leaves, roots, and rhizoplane soil sources (Figure 4b). The strains that tested strongly positive for cyanogenesis were mainly from the genus *Pseudomonas*, and to a lesser extent from the genera *Microbacterium* and *Pantoea*.

Twenty‐three of the 84 tested strains were able to grow in media supplemented with ACC as the sole nitrogen source confirming their ability to produce ACC deaminase (Figure 4c). The majority of these positive strains were Gram‐negative bacteria belonging to the genera *Pseudomonas*,* Pantoea*, and *Enterobacter*. These strains that tested positive for ACC deaminase activity were also positive for multiple biochemical properties.

Forty‐seven strains were capable of solubilizing phosphates (Figure 4d). This characteristic was evenly distributed in the bacterial strains isolated from the three different sources. Fifty‐seven of the tested strains were positive for siderophore production. The distribution of this trait was regardless of genera and isolation sources. However, the majority of the strains that tested positive for siderophoregenesis were from the genus *Pseudomonas* and were isolated from rhizoplane soil. Most of the strains that were negative for siderophoregenesis were from roots and leaves (Figure 4e).

### 3.4 Antifungal activity

Some bacterial strains were detected with antifungal activity against *Aspergillus flavus* (F006), *Aspergillus flavus* (F023), *Nigrospora oryzae* (F025), *Magnaporthe grisea* (MG001), and *Nigrospora sphaerica* (F010). No bacterial strain showed antifungal activity against *Fusarium equiseti* (F005) and *Phoma herbarum* (F020). A total of 4, 2, 15, 12, and 1 strain showed antifungal activity against *A. flavus* (F006), *A. flavus* (F023), *N. oryzae* (F025), *N. sphaerica* (F010), and *M. grisea* (MG001), respectively. Some strains, for example, *Pseudomonas s*pp. (CSB_B072) and *Pectobacterium carotovorum* (CSB_B046) showed antifungal activity against four and three pathogenic fungi, respectively (Figure 4Fi‐vii). Seventy‐nine percent of bacterial strains that tested positive for antifungal activity were from roots and rhizoplane soil.

**Figure 5:**
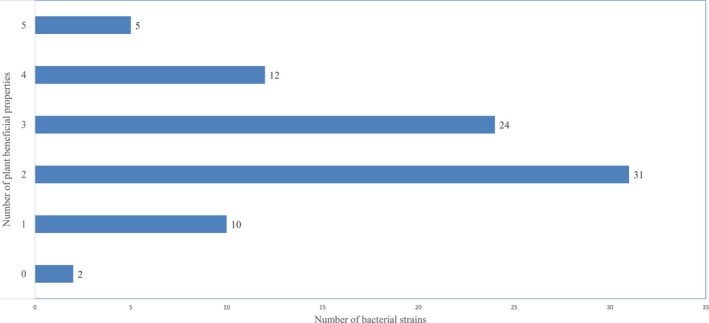
Screening endophytic and plant‐associated bacteria of Brachiaria grasses for plant beneficial properties. Strains were tested for auxin production, cyanogenesis, ACC‐deaminase production, phosphate solubilization, siderophoregenesis, and antifungal activities

Evaluations of the 84 bacterial strains isolated from Brachiaria grasses and rhizoplane soil for six different biochemical tests showed them having up to five properties beneficial to plant growth and development (Figure 5). Over 97% of tested strains were positive to one or more of the six biochemical tests.

### 3.5 Plant growth promotion

**Figure 6:**
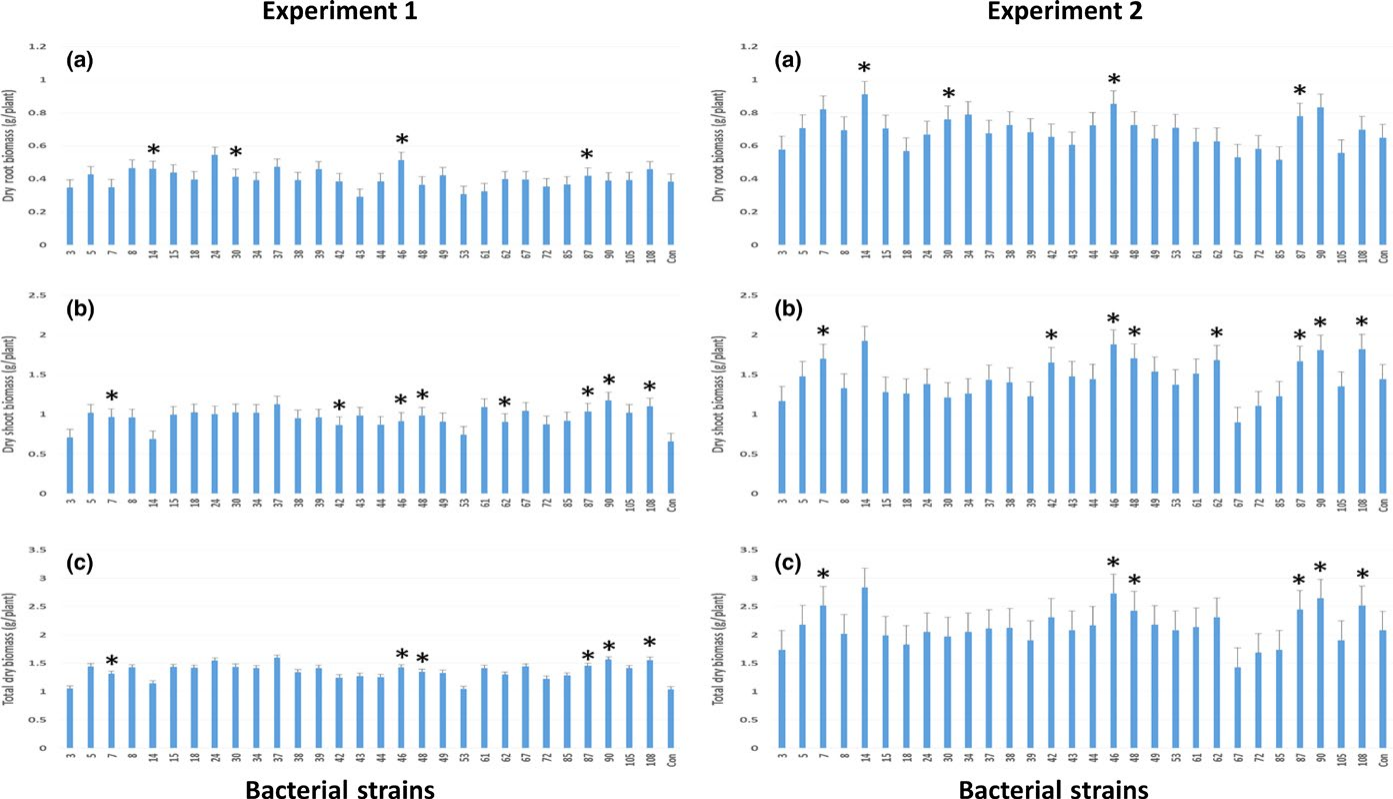
Effect of bacterial inoculation on root (a), shoot (b), and total biomass (c) of maize seedlings. Four, seven, and six bacterial strains (indicated with asterisk) significantly (*p < *.05) increased root, shoot, and total dry biomass production, respectively, in both experiments. Test bacterial strain IDs in the figure are presented without prefix CSB_B, strain 7 was included as positive control, and “Con” represent control plants with no bacteria inoculation. Error bars represent standard error of mean

Twenty‐nine bacterial strains evaluated in this study had variable effects on root and shoot biomass production of maize seedlings (Figure 6). Irrespective of the treatment shoot and root biomass production was higher in the experiment 2 than those recorded from experiment 1 (*p < *.05). Four bacteria strains significantly increased root biomass in both experiments, whereas eight strains had similar effect on shoot biomass. A total of six tested bacterial strains (CSB_B007, CSB_B046, CSB_B048, CSB_B087, CSB_B090, and CSB_B108) had consistently significant (*p ≤ *.05) positive effect on total biomass production. The best performing strain (CSB_B090) increased the total biomass production by as high as 39% (Figure 6). Interesting to note is that two strains (CSB_B046 and CSB_B087) had positive effect on both shoot and root biomass production of maize seedlings.

## 4 DISCUSSION

We isolated 84 bacterial strains from the leaves, roots, and rhizoplane soil of Brachiaria grasses growing in wild at the International Livestock Research Institute in Nairobi, Kenya. The identity of these bacterial strains was established based on 16S rDNA sequences and relationships among the strains were established through phylogenetic analysis; with 50 unique sequences identified among the test strains as representative of the 84 isolated strains. The analysis grouped the 50 representative bacterial strains into 7 distinct clades. Biochemical characterization of bacterial strains revealed that over 97% of tested strains were positive for one to five of six biochemical tests demonstrating significant roles of these microbes in plant growth and development.

This study focused on isolation, identification, and characterization of cultivable endophytes and rhizoplane soil bacteria for potential agricultural applications. The bacterial communities of Brachiaria grasses were composed of many closely related strains, the majority of which belong to the Phylum *Gammaproteobacteria*. The dominance of *Gammaproteobacteria* (73%) in Brachiaria grasses is comparable to that reported for poplar and willow, the bacteria from tree peony rhizoplane, as well as the maize rhizosphere (García‐Salamanca et al., 2012; Han, Song, Liu, & Hu, 2011; Moore et al., 2006; Taghavi et al., 2009). *Gammaproteobacteria* respond chemotactically to root exudates and are very efficient in utilizing plant exudates (García‐Salamanca et al., 2012), therefore, they are abundant in rhizoplane soils as well as in roots and leaves samples. *Gammaproteobacteria* of genera *Pseudomonas, Pantoea*, and *Acinetobacter* constituted 27%, 20%, and 11% of the microbial populations, respectively, and were consistently isolated from rhizoplane soil, roots, and leaves. The root colonization by bacteria is viewed as a continuum from rhizosphere to rhizoplane to internal root tissues, with some bacteria capable of going beyond the endodermis, to pass through root cortex to the vascular system and subsequently reach aboveground plant tissues (Hallmann, Rodrıguez‐Kábana, & Kloepper, 1999).

Analysis of the 16S rDNA sequences of 84 bacterial strains revealed 50 OTUs belonging to 16 genera at different frequencies that ranged from 1 to 23. This observation is comparable to similar studies on poplar and willow (Moore et al., 2006; Taghavi et al., 2009). The number of bacterial genera isolated from rhizoplane soil, roots, and leaves of Brachiaria was 5, 12, and 8, respectively. As anticipated, bacteria from roots were more diverse than those from leaves, whereas rhizoplane soil had the least diversity at genus level. A limited diversity in rhizoplane soil bacteria might have been attributed to the high affinity between selected bacterial species and root exudates, and abundance of these bacteria interfering with the recovery of other bacteria that are present in low numbers.

Phylogenetic analysis of the 50 representative OTUs revealed them into seven distinct clades; with all but two clades (clades VI and VII) dominated by members of the Phylum Proteobacteria. This is in agreement with the fact that Proteobacteria are morphologically, physiologically, and ecologically extremely diverse, and is one of the largest Phyla accounting for over 45% of all cultured bacteria (Kersters et al., 2006).

Endophytes and rhizobacteria are part of the natural microflora of healthy plants and may therefore be considered to be important contributors to plant growth and biological control of pathogens and weeds (Hallmann, Quadt‐Hallmann, Mahaffee, & Kloepper, 1997). Fifty‐eight percent of the bacterial strains isolated in this study were able to produce the IAA. The production of IAA has been reported for many bacteria and it is assumed that over 80% of the bacteria isolated from the rhizosphere are capable of synthesizing IAA (Khalid, Tahir, Arshad, & Zahir, 2004; Patten & Glick, 1996). Auxin plays a major role in the regulation of various plant physiological processes such as cell division and enlargement, cell differentiation, and cellular response to physical factors like light and gravity (Bartel, 1997; Meuwly & Pilet, 1991). The level of IAA in a plant has an effect on primary root length and formation of adventitious and lateral roots and this consequently influences water and nutrient uptake. A number of plant‐associated bacteria have been shown to have the ability to produce IAA and contribute to plant growth promotion by altering the plant auxin pool (Bharucha, Patel, & Trivedi, 2013).

Twenty six percent of the tested bacterial strains were able to produce hydrogen cyanide. The production of HCN by plant‐associated microorganisms has been demonstrated as one of the mechanisms for biological control of weeds, nematodes and microbial pathogens (Kremer and Souissi, 2001). In bacteria, cyanogenesis has been reported mainly in the genus Pseudomonas (Ryall et al., 2009), a few bacilli (Grover et al., 2009) and members of the Burkholderia cepacia complex (Ryall et al., 2008).

Twenty seven percent of the isolated bacterial strains were found to possess 1‐aminocyclopropane‐1‐carboxylate (ACC) deaminase activity. The hormone ethylene plays a role in various physiological processes in plants, including the breaking of seed dormancy, but sustained high levels in response to biotic and abiotic stress can inhibit root growth and induce senescence (Akhgar et al., 2014). Endophytic bacteria that can produce the enzyme ACC deaminase contribute towards the catabolism of the plant ethylene precursor, ACC, consequently decreasing plant ethylene level and enabling plants to better tolerate biotic and abiotic stresses (Glick et al., 1998, Glick et al., 2007). Microbial ability to produce ACC deaminase has been identified as one of the direct mechanisms of plant growth‐promotion and has been linked to drought and salt tolerance in various plant species (Akhgar et al., 2014; Glick, 2005; Sgroy et al., 2009).

About 56% of tested bacterial strains were able to solubilize phosphorous. Phosphorus is an important element that often occurs in abundance in soils but its availability to plants is limited because it occurs mainly in the form of insoluble complexes that cannot be taken up by plants (Goldstein, 1986). Phosphate solubilizing microorganisms have the ability to convert inorganic and organic phosphate complexes into bioavailable forms that can easily be taken up by plants therefore promoting plant growth (Hilda and Fraga, 1999; Turan et al., 2006). The use of phosphate solubilizing microbes is a sustainable approach for managing phosphorus deficiency in agricultural soils.

More than half (51%) of the isolated bacterial strains tested positive for siderophore production. Siderophores are low molecular weight iron‐chelating agents secreted by bacteria in iron‐limiting conditions to help them scavenge for iron from the environment (Neilands and Nakamura, 1991; Neilands, 1981).

The production of siderophores by plant‐associated microorganisms stimulates plant growth by depriving plant pathogens of iron which inhibits the growth of such pathogens and also by making iron available to the plants (Costa & Loper, 1994).

Some of the tested strains showed antifungal activities against five plant pathogenic fungi representing three genera. Antifungal activity has been demonstrated in several genera of bacteria (Kerr et al., 1999) with iron deprivation through siderophores, cyanogenesis, and antibiosis through the secretion of enzymes and volatile compounds described as some of the possible mechanisms through which such bacteria effect antifungal activity (Cornelison, Gabriel, Barlament, & Crow, 2014; Frey‐Klett et al., 2011; Minaeva, Akimova, & Evdokimov, 2008).

This study shows that Brachiaria grasses host diverse groups of bacteria that are beneficial to plant growth promotion and suppression of plant pathogens. It is impressive that over 97% of bacterial strains isolated from Brachiaria tissues and rhizoplane soil had one or more plant beneficial properties. More fascinating was that 41 of 84 bacterial strains were positive for three or more plant beneficial traits. A subset of these bacterial strains when tested for plant growth promotion on maize seedlings, six tested strains significantly *(p *≤* *.05) increased total biomass production as reported in poplar (Taghavi et al., 2009). The results presented in this study are based on the culture‐dependent approach in consistence with the aim of this study; to identify microbes for agricultural applications. A full understanding of microbial community of Brachiaria grasses requires the complementation of this work with culture independent approaches that uses advanced genomic and bioinformatics tools (Donn et al.*,* 2015; Mao, Li, Smyth, Yannarell, & Mackie, 2014; Peiffer et al., 2013).

Brachiaria are extensively cultivated tropical forage grasses known for several desirable qualities including drought tolerance; adaptation to low fertility soils; high nitrogen use efficiency, less input demand, high biomass production; carbon sequestration; and resistance to several pests and diseases. Some of these outstanding attributes of Brachiaria grasses could be associated with the microbes they harbor, endophytically, and within its surrounding environment, that is, rhizoplane and rhizosphere. What makes Brachiaria grasses so successful even under apparently harsh environmental and low input conditions has been the subject of speculation to many researchers. Our current findings on bacterial community composition and the beneficial traits these microbes hold have furnished some evidence on the potential role of these microbes on forage biomass production and the adaptation of Brachiaria grasses to drought and low fertility soil. Further work on evaluation of subset of these bacterial strains for plant growth and development, adaptation to drought and low fertility soils in the greenhouse and field environments are suggested toward developing microbial‐based low input technology for the forage production in sub‐Saharan Africa.

## CONFLICT OF INTEREST

The authors declare that there is no conflict of interest regarding the publication of this study.

## References

[mbo3497-bib-0002] Ademosun, A. A. (1976). Livestock Production in Nigeria: Our Commission and Omission. Ile‐Ife Nigeria: University of Ife Press.

[mbo3497-bib-0003] Akhgar, A. R. , Arzanlou, M. , Bakker, P. A. H. M. , & Hamidpour, M . (2014). Characterization of 1‐Aminocyclopropane‐1‐Carboxylate (ACC) deaminase‐containing *Pseudomonas* spp. in the Rhizosphere of salt‐stressed canola. Pedosphere, 24 (4), 461–468.

[mbo3497-bib-0004] Ali, S. Z. , Sandhya, V. , & Rao, L. V. (2014). Isolation and characterization of drought‐tolerant ACC deaminase and exopolysaccharide‐producing fluorescent *Pseudomonas* sp. Annals of Microbiology, 64(2), 493–502.

[mbo3497-bib-0005] Alstrom, S. , & Burns, R. G. (1989). Cyanide production by rhizobacteria as a possible mechanism of plant growth inhibition. Biology and Fertility of Soils, 7, 232–237.

[mbo3497-bib-0006] Bahulikar, R. A. , Torres‐Jerez, I. , Worley, E. , Craven, K. D. , & Udvardi, M. K. (2014). Diversity of nitrogen‐fixing bacteria associated with switchgrass in the native tall‐grass prairie of northern Oklahoma. Applied and Environmental Microbiology, 80(18), 5636–5643. 25002418 10.1128/AEM.02091-14 PMC4178587

[mbo3497-bib-0007] Bartel, B. (1997). Auxin biosynthesis. Annual Review of Plant Physiology and Plant Molecular Biology, 48, 51–66. 10.1146/annurev.arplant.48.1.51 15012256

[mbo3497-bib-0008] Bharucha, U. , Patel, K. , & Trivedi, U. B. (2013). Optimization of Indole Acetic Acid Production by Pseudomonas putida UB1 and its Effect as Plant Growth‐Promoting Rhizobacteria on Mustard (*Brassica nigra*). Agriculture Research, 2(3), 215–221.

[mbo3497-bib-0009] Cole, J. R. , Wang, Q. , Fish, J. A. , Chai, B. , McGarrell, D. M. , Sun, Y. , … Tiedje, J. M . (2014). Ribosomal Database Project: Data and tools for high throughput rRNA analysis. Nucleic Acids Research, 42 (Database issue):D633–D642. 24288368 10.1093/nar/gkt1244 PMC3965039

[mbo3497-bib-0010] Compant, S. , Duffy, B. , Nowak, J. , Clément, C. , & Barka, E. A. (2005). Use of Plant Growth‐Promoting Bacteria for Biocontrol of Plant Diseases: Principles, Mechanisms of Action, and Future Prospects. Applied and Environmental Microbiology, 71(9), 4951–4959. 16151072 10.1128/AEM.71.9.4951-4959.2005 PMC1214602

[mbo3497-bib-0011] Cornelison, C. T. , Gabriel, K. T. , Barlament, C. , & Crow, S. A. (2014). Inhibition of *Pseudogymnoascus destructans* Growth from Conidia and Mycelial Extension by Bacterially Produced Volatile Organic Compounds. Mycopathologia, 177(1–2), 1–10. 24190516 10.1007/s11046-013-9716-2

[mbo3497-bib-0012] Costa, J. M. , & Loper, J. E. (1994). Characterization of Siderophore Production by the Biological Control Agent *Enterobacter cloacae* . Molecular Plant‐Microbe Interactions, 7(4), 440–448.

[mbo3497-bib-0013] Davison, J. (1988). Plant beneficial bacteria. Nature Biotechnology, 6, 282–286.

[mbo3497-bib-0014] Donn, S. , Kirkegaard, J. A. , Perera, G. , Richardson, A. E. , & Watt, M. (2015). Evolution of bacterial communities in the wheat crop rhizosphere. Environmental Microbiology, 17(3), 610–621. 24628845 10.1111/1462-2920.12452

[mbo3497-bib-0015] FAO . (2016). FAO's role in livestock and the environment. (http://www.fao.org/livestock-environment/en/). Accessed on 20 October, 2016.

[mbo3497-bib-0016] Fisher, M. J. , & Kerridge, P. C . (1996). The agronomy and physiology of Brachiaria species In J. W. Miles, B. L. Maass, C. B. do Valle, V. Kumble (eds), Brachiaria: Biology, Agronomy, and Improvement (pp. 43–52). Cali, Colombia: International Center for Tropical Agriculture.

[mbo3497-bib-0017] Fisher, M. J. , Rao, I. M. , Ayarza, M. A. , Lascano, C. , Sanz, J. I. , Thomas, R. J. , & Vera, R. R. (1994). Carbon storage by introduced deep‐rooted grasses in the South American Savannas. Nature, 371, 236–238.

[mbo3497-bib-0018] Frank, J. A. , Reich, C. I. , Sharma, S. , Weisbaum, J. S. , Wilson, B. A. , & Olsen, G. J. (2008). Critical Evaluation of Two Primers Commonly Used for Amplification of Bacterial 16S rRNA Genes. Applied and Environmental Microbiology, 74, 2461–2470. 18296538 10.1128/AEM.02272-07 PMC2293150

[mbo3497-bib-0019] Frey‐Klett, P. , Burlinson, P. , Deveau, A. , Barret, M. , Tarkka, M. , & Sarniguet, A. (2011). Bacterial‐Fungal Interactions: Hyphens between Agricultural, Clinical, Environmental, and Food Microbiologists. Microbiology and Molecular Biology Review, 75(4), 583–609. 10.1128/MMBR.00020-11 PMC3232736 22126995

[mbo3497-bib-0020] García de Salamone, I. E. , Hynes, R. K. , & Nelson, L. M. (2001). Cytokinin production by plant growth promoting rhizobacteria and selected mutants. Canadian Journal of Microbiology, 47(5), 404–411. 11400730 10.1139/w01-029

[mbo3497-bib-0021] García‐Salamanca, A. , Molina‐Henares, M. A. , van Dillewijn, P. , Solano, J. , Pizarro‐Tobías, P. , Roca, A. , … Ramos, J. L. (2012). Bacterial diversity in the rhizosphere of maize and the surrounding carbonate‐rich bulk soil. Microbial Biotechnology, 6, 36–44. 22883414 10.1111/j.1751-7915.2012.00358.x PMC3815383

[mbo3497-bib-0022] Glick, B. R. (2005). Modulation of plant ethylene levels by the bacterial enzyme ACC deaminase. FEMS Microbiology Letters, 251(1), 1–7. 16099604 10.1016/j.femsle.2005.07.030

[mbo3497-bib-0023] Glick, B. R. , Cheng, Z. , Czarny, J. , & Duan, J. (2007). Promotion of plant growth by ACC deaminase‐containing soil bacteria. European Journal of Plant Pathology, 119, 329–339.

[mbo3497-bib-0024] Glick, B. R. , Penrose, D. M. , & Li, J. (1998). A model for the lowering of plant ethylene concentrations by plant growth promoting bacteria. Journal of Theoretical Biology, 190, 63–68. 9473391 10.1006/jtbi.1997.0532

[mbo3497-bib-0025] Goddijn, O. , & Smeekens, S. (1998). Sensing trehalose biosynthesis in plants. Plant Journal, 14(2), 143–146. 9628011 10.1046/j.1365-313x.1998.00140.x

[mbo3497-bib-0026] Goldstein, A. H. (1986). Bacterial solubilization of mineral phosphates: Historical perspectives and future prospects. American Journal of Alternative Agriculture, 1(02), 51–57.

[mbo3497-bib-0027] Grover, M. , Nain, L. , & Saxena, A. K. (2009). Comparison between *Bacillus subtilis* RP24 and its antibiotic‐defective mutants. World Journal of Microbiology and Biotechnology, 25, 1329–1335.

[mbo3497-bib-0028] Gutierrez‐Manero, F. J. , Ramos‐Solano, B. , Probanza, A. , Mehouachi, J. , Tadeo, F. R. , & Talon, M. (2001). The plant growth‐promoting rhizobacteria *Bacillus licheniformis* produce high amounts of physiologically active gibberillins. Physiologia Plantarum, 111, 206–211.

[mbo3497-bib-0029] Hallmann, J. , Quadt‐Hallmann, A. , Mahaffee, W. F. , & Kloepper, J. W. (1997). Bacterial endophytes in agricultural crops. Canadian Journal of Microbiology, 43, 895–914.

[mbo3497-bib-0030] Hallmann, J. , Rodrıguez‐Kábana, R. , & Kloepper, J. W. (1999). Chitin‐mediated changes in bacterial communities of the soil, rhizosphere and within roots of cotton in relation to nematode control. Soil Biology and Biochemistry, 31(4), 551–560.

[mbo3497-bib-0031] Han, J. , Song, Y. , Liu, Z. , & Hu, Y. (2011). Culturable bacterial community analysis in the root domains of two varieties of tree peony (*Paeonia ostii*). FEMS Microbiology Letters, 322(1), 15–24. 21623895 10.1111/j.1574-6968.2011.02319.x

[mbo3497-bib-0032] Hilda, R. , & Fraga, R. (1999). Phosphate solubilizing bacteria and their role in plant growth promotion. Biotechnology Advances, 17, 319–339. 14538133 10.1016/s0734-9750(99)00014-2

[mbo3497-bib-0033] James, E. K. (2000). Nitrogen fixation in endophytic and associative symbiosis. Field Crop Research, 65, 197–209.

[mbo3497-bib-0034] Jank, L. , Barrios, S. C. , do Valle, C. B. , Simeão, R. M. , & Alves, G. F . (2014). The value of improved pastures to Brazilian beef production. Crop and Pasture Science, 65 (11), 1132–1137.

[mbo3497-bib-0035] Kelemu, S. , Fory, P. , Zuleta, C. , Ricaurte, J. , Rao, I. , & Lascano, C. (2011). Detecting bacterial endophytes in tropical grasses of the Brachiaria genus and determining their role in improving plant growth. African Journal of Biotechnology, 10(6), 965–976.

[mbo3497-bib-0036] Kerr, J. R. , Taylor, G. W. , Rutman, A. , Høiby, N. , Cole, P. J. , & Wilson, R. (1999). *Pseudomonas aeruginosa* pyocyanin and 1‐hydroxyphenazine inhibit fungal growth. Journal of Clinical Pathology, 52(5), 385–387. 10560362 10.1136/jcp.52.5.385 PMC1023078

[mbo3497-bib-0037] Kersters, K. , De Vos, P. , Gillis, M. , Swings, J. , Vandamme, P. , & Stackebrandt, E . (2006). Introduction to the Proteobacteria In Dworkin M., Falkow S., Rosenberg E., Schleifer K. H. & Stackebrandt E. (eds.), The Prokaryotes: A Handbook on the Biology of Bacteria (3rd ed.), Proteobacteria: Alpha and Beta Subclasses (vol. 5) (pp. 3–37). New York, Springer, Release 3.12 edn.

[mbo3497-bib-0038] Khalid, A. , Tahir, S. , Arshad, M. , & Zahir, Z. A. (2004). Relative efficiency of rhizobacteria for auxin biosynthesis in rhizosphere and non‐rhizosphere soils. Australian Journal of Soil Research, 42, 921–926.

[mbo3497-bib-0039] Kloepper, J. W. , Leong, J. , Teintze, M. , & Schroth, M. N. (1980). Enhanced plant growth by siderophores produced by plant growth‐promoting rhizobacteria. Nature, 286, 885–886.

[mbo3497-bib-0040] Kremer, R. J. , & Souissi, T. (2001). Cyanide production by rhizobacteria and potential for suppression of weed seedling growth. Current Microbiology, 43(3), 182–186. 11400067 10.1007/s002840010284

[mbo3497-bib-0041] Lambert, B. , & Joos, H. (1989). Fundamental aspects of rhizobacterial plant growth promotion research. Trends in Biotechnology, 7, 215–219.

[mbo3497-bib-0042] Lorck, H. (1948). Production of hydrocyanic acid by bacteria. Physiologia Plantarum, 1(2), 142–146.

[mbo3497-bib-0043] Maass, B. L. , Midega, A. O. , Mutimura, M. , Rahetlah, V. B. , Salgado, P. , Kabirizi, J. M. , … Rao, I. M. (2015). Homecoming of Brachiaria: Improved hybrids prove useful for African animal agriculture. East African Agricultural and Forestry Journal, 81(1), 71–78.

[mbo3497-bib-0044] Mao, Y. , Li, X. , Smyth, E. M. , Yannarell, A. C. , & Mackie, R. I. (2014). Enrichment of specific bacterial and eukaryotic microbes in the rhizosphere of switchgrass (*Panicum virgatum* L.) through root exudates. Environmental microbiology reports, 6 (3), 293–306. 24983534 10.1111/1758-2229.12152

[mbo3497-bib-0045] Mastretta, C. , Barac, T. , Vangronsveld, J. , Newman, L. , Taghavi, S. , & van der Lelie, D. (2006). Endophytic Bacteria and their Potential Application to Improve the Phytoremediation of Contaminated Environments. Biotechnology and Genetic Engineering Reviews, 23, 175–188. 22530508 10.1080/02648725.2006.10648084

[mbo3497-bib-0046] Mayer, A. (1958). Determination of indole acetic acid by Salkowsky reaction. Nature, 182, 1670–1671. 13622611 10.1038/1821670a0

[mbo3497-bib-0047] Mehta, S. , & Nautiyal, C. S. (2001). An efficient method for qualitative screening of phosphate‐solubilizing bacteria. Current Microbiology, 43, 51–56. 11375664 10.1007/s002840010259

[mbo3497-bib-0048] Mendes, R. , Kruijt, M. , de Bruijn, I. , Dekkers, E. , van der Voort, M. , Schneider, J. H. M. , … Raaijmakers, J. M. (2011). Deciphering the Rhizosphere Microbiome for Disease‐Suppressive Bacteria. Science, 332, 1097–1100. 21551032 10.1126/science.1203980

[mbo3497-bib-0049] Meuwly, P. , & Pilet, P. E. (1991). Local treatment with indole‐3‐acetic acid induces differential growth responses in *Zea mays* L. roots. Planta, 185, 58–64. 24186280 10.1007/BF00194515

[mbo3497-bib-0050] Minaeva, O. M. , Akimova, E. L. , & Evdokimov, E. V. (2008). Kinetic aspects of inhibition of the phytopathogenic fungi growth by rhizosphere bacteria. Applied Biochemistry and Microbiology, 44(5), 512–517. 18822777

[mbo3497-bib-0051] Moore, F. P. , Barac, T. , Borremans, B. , Oeyen, L. , Vangronsveld, J. , van der Lelie, D. , … Moore, E. R. B. (2006). Endophytic bacterial diversity in poplar trees growing on a BTEX‐contaminated site: The characterisation of isolates with potential to enhance phytoremediation. Systematic and Applied Microbiology, 29, 539–556. 16919907 10.1016/j.syapm.2005.11.012

[mbo3497-bib-0052] Neilands, J. B. (1981). Iron absorption and transport in microorganisms. Annual Review of Nutrition, 1, 27–46. 10.1146/annurev.nu.01.070181.000331 6226306

[mbo3497-bib-0053] Neilands, J. B. , & Nakamura, K . (1991). Detection, determination, isolation, characterization and regulation of microbial iron chelates In Winkelmann G. (Eds.), CRC Handbook of Microbial Iron Chelate (pp. 1–14). Boca Raton, Florida: CRC Press.

[mbo3497-bib-0054] Otieno, N. , Lally, R. D. , Kiwanuka, S. , Lloyd, A. , Ryan, D. , Germaine, K. J. , & Dowling, D. N. (2015). Plant Growth Promotion Induced by Phosphate Solubilizing Endophytic Pseudomonas Isolates. Frontiers in Microbiology, 6, 745 https://doi.org/10.3389/fmicb.2015.00745 26257721 10.3389/fmicb.2015.00745 PMC4510416

[mbo3497-bib-0055] Patten, C. L. , & Glick, B. R. (1996). Bacterial biosynthesis of indole‐3‐acetic acid. Canadian Journal of Microbiology, 42, 207–220. 8868227 10.1139/m96-032

[mbo3497-bib-0056] Peiffer, J. A. , Spor, A. , Koren, O. , Jin, Z. , Tringe, S. G. , Dangl, J. L. , … Ley, R. E. (2013). Diversity and heritability of the maize rhizosphere microbiome under field conditions. Proceedings of the National Academy of Sciences, 110, 6548–6553. 10.1073/pnas.1302837110 PMC3631645 23576752

[mbo3497-bib-0057] Rao, I. M. , Kerridge, P. C. , & Macedo, M. C. M . (1996). Nutritional requirements of Brachiaria and adaptation to acid soils In Miles J. W., Maass B. L., do Valle C. B. & Kumble V. (eds.). Brachiaria: Biology, Agronomy, and Improvement (pp. 53–71). International Center for Tropical Agriculture: Cali, Colombia.

[mbo3497-bib-0058] Rungin, S. , Indananda, C. , Suttiviriya, P. , Kruasuwan, W. , Jaemsaeng, R. , & Thamchaipenet, A. (2012). Plant growth enhancing effects by a siderophore‐producing endophytic streptomycete isolated from a Thai jasmine rice plant (*Oryza sativa* L. cv. KDML105). Antonie van Leeuwenhoek, 102, 463–472. 22836676 10.1007/s10482-012-9778-z

[mbo3497-bib-0059] Ryall, B. , Lee, X. , Zlosnik, J. E. , Hoshino, S. , & Williams, H. D. (2008). Bacteria of the *Burkholderia cepacia* complex are cyanogenic under biofilm and colonial growth conditions. BMC Microbiology, 8, 108. 18588687 10.1186/1471-2180-8-108 PMC2504479

[mbo3497-bib-0060] Ryall, B. , Mitchell, H. , Mossialos, D. , & Williams, H. D. (2009). Cyanogenesis by the entomopathogenic bacterium *Pseudomonas entomophila* . Letters in Applied Microbiology, 49, 131–135. 19486283 10.1111/j.1472-765X.2009.02632.x

[mbo3497-bib-0061] Sgroy, V. , Cassán, F. , Masciarelli, O. , Del Papa, M. F. , Lagares, A. , & Luna, V. (2009). Isolation and characterization of endophytic plant growth‐promoting (PGPB) or stress homeostasis‐regulating (PSHB) bacteria associated to the halophyte *Prosopis strombulifera* . Applied Microbiology and Biotechnology, 85(2), 371–381. 19655138 10.1007/s00253-009-2116-3

[mbo3497-bib-0062] Subbarao, G. V. , Nakahara, K. , Hurtado, M. P. , Ono, H. , Moreta, D. E. , Salcedo, A. F. , … Ito, O. (2009). Evidence for biological nitrification inhibition in Brachiaria pastures. Proceedings of the National Academy of Sciences of the United States of America, 106, 17302–17307. 19805171 10.1073/pnas.0903694106 PMC2752401

[mbo3497-bib-0063] Taghavi, S. , Garafola, C. , Monchy, S. , Newman, L. , Hoffman, A. , Weyen, N. , … van der Lelie, D. (2009). Genome survey and characterization of endophytic bacteria exhibiting a beneficial effect on growth and development of poplar trees. Applied and Environmental Microbiology, 75, 748–757. 19060168 10.1128/AEM.02239-08 PMC2632133

[mbo3497-bib-0064] Taghavi, S. , Van Der Lelie, D. , Hoffman, A. , Zhang, Y. B. , Walla, M. D. , Vangronsveld, J. , … Monchy, S. (2010). Genome sequence of the plant growth promoting endophytic bacterium *Enterobacter sp*. 638. PLoS Genetics, 6(5), e1000943. 20485560 10.1371/journal.pgen.1000943 PMC2869309

[mbo3497-bib-0065] Tamura, K. , Stecher, G. , Peterson, D. , Filipski, A. , & Kumar, S. (2013). MEGA6: Molecular Evolutionary Genetics Analysis version 6.0. Molecular Biology and Evolution, 30, 2725–2729. 24132122 10.1093/molbev/mst197 PMC3840312

[mbo3497-bib-0066] Turan, M. , Ataoglu, N. , & Sahin, F. (2006). Evaluation of the capacity of phosphate solubilizing bacteria and fungi on different forms of phosphorus in liquid culture. Journal of Sustainable Agriculture, 28, 99–108.

[mbo3497-bib-0501] Turan, M. , Gulluce, M. , von Wirén, N. , & Sahin, F. (2012). Yield promotion and phosphorus solubilization by plant growth–promoting rhizobacteria in extensive wheat production in Turkey. Plant nutrition and soil science, 175, 818–826.

[mbo3497-bib-0067] van der Lelie, D. , Taghavi, S. , Monchy, S. , Schwende, J. , Miller, L. , Ferrieri, R. , … Newman, L. (2009). Poplar and its Bacterial Endophytes: Coexistence and Harmony. Critical Reviews in Plant Sciences, 28, 346–358.

[mbo3497-bib-0068] Vellore, J. M . (2001). Iron acquisition in Rhodococcus erythrolpolis: the isolation of mutant(s) that do not produce a siderophore. Electronic Theses and Dissertations. Paper 41. http://dc.etsu.edu/etd/41

[mbo3497-bib-0069] VSN International . (2013). GenStat for Windows (15th Edition), Hemel Hempstead, UK: VSN International Web page: https://www.vsni.co.uk/software/genstat/

[mbo3497-bib-0070] Wu, F. , Wan, J. H. C. , Shengchun Wu, S. K. , & Wong, M . (2012). Effects of earthworms and plant growth–promoting rhizobacteria (PGPR) on availability of nitrogen, phosphorus, and potassium in soil. Journal of Plant Nutrition and Soil Sciences, 175, 423–433.

[mbo3497-bib-0071] Zhang, Y. F. , He, L. Y. , Chen, Z. J. , Wang, Q. Y. , Qian, M. , & Sheng, X. F. (2011). Characterization of ACC deaminase‐producing endophytic bacteria isolated from copper‐tolerant plants and their potential in promoting the growth and copper accumulation of *Brassica napus* . Chemosphere, 83, 57–62. 21315404 10.1016/j.chemosphere.2011.01.041

