# Supplementary figures and images for: Brachiaria Grasses (Brachiaria spp.) harbor a diverse bacterial community with multiple attributes beneficial to plant growth and development
Source: Microbiologyopen. 2017 Jun 21;6(5):e00497. doi: 10.1002/mbo3.497 (PMC5635169; doi:10.1002/mbo3.497)

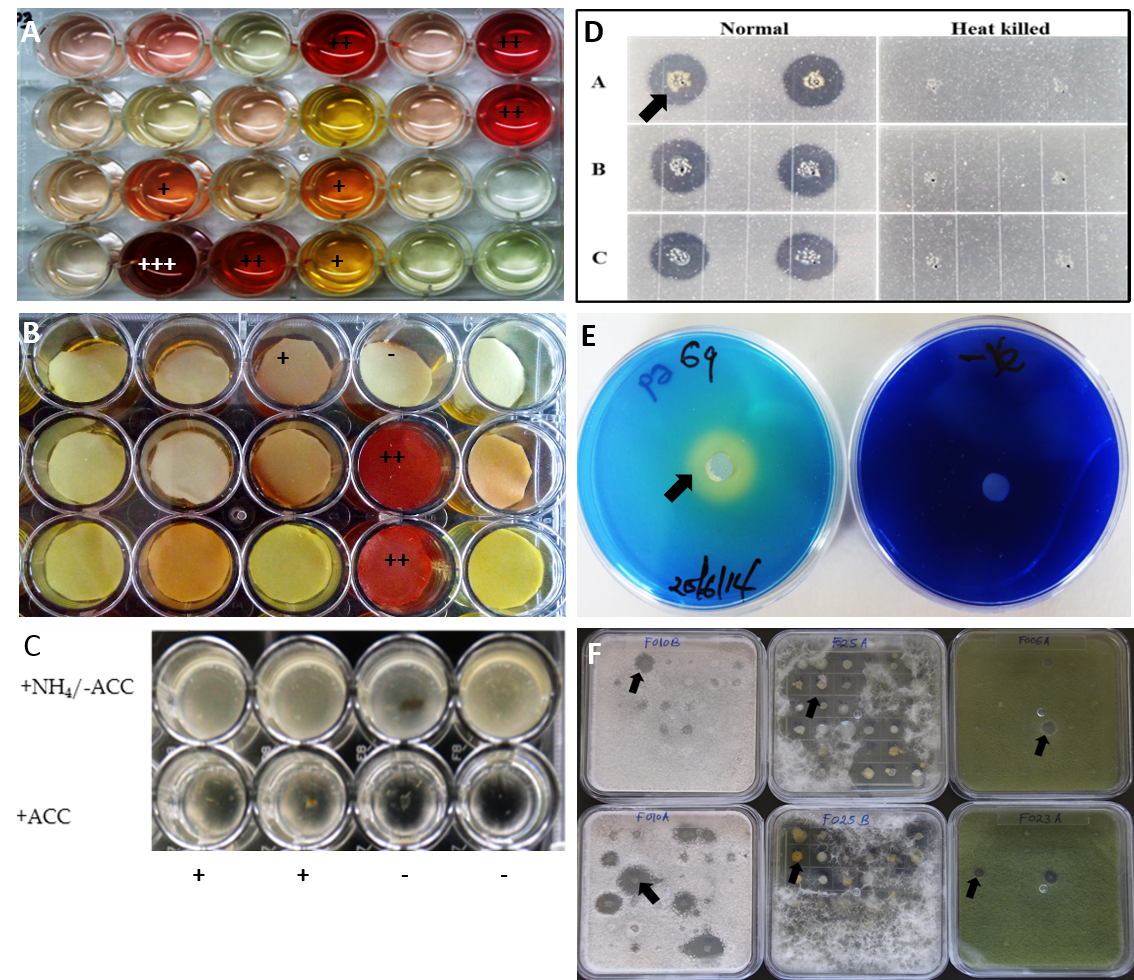

Supplement: Supplementary file 1 [file MBO3-6-na-s001.tif]
